# Precision of an automated surface-based CT-RSA (asCTRSA) for assessing tibial implant migration: a porcine cadaver study and a clinical case

**DOI:** 10.2340/17453674.2026.46156

**Published:** 2026-07-16

**Authors:** Manou ACKE, Benyameen KEELSON, Lars H W ENGSETH, Frank-David ØHRN, Gert VAN GOMPEL, Stephan M RÖHRL, Anselm SCHULZ, Johan DE MEY, Nico BULS

**Affiliations:** 1 Department of Radiology, Vrije Universiteit Brussel (VUB), Universitair Ziekenhuis Brussel (UZ Brussel), Brussels, Belgium;; 2 Faculty of Medicine, University of Oslo (UiO), Oslo, Norway; 3 Division for Orthopedic Surgery, Oslo University Hospital (OUH), Oslo, Norway; 4 Nordmøre and Romsdal Hospital (SNR), Møre and Romsdal Hospital Trust, Hjelsetay, Norway; 5 Department of Neuromedicine and Movement Science (INB), Faculty of Medicine and Health Sciences, Norwegian University of Science and Technology (NTNU), Trondheim, Norway; 6 Akershus University Hospital (Ahus), Department of Radiology, Lørenskog, Norway

## Abstract

**Background and purpose:**

Radiostereometric analysis (RSA) is the reference standard for migration analysis, but adoption is limited by invasive marker implantation and specialized equipment. We developed an automated, surface-based CT-RSA (asCTRSA) method that reports a registration quality measure (mean rigid-body fitting error, ME). Our aim was to evaluate the precision by comparing asCTRSA with marker-based RSA and CT-based Micromotion Analysis (CTMA, v25.1).

**Methods:**

Precision was assessed in a retrospective, repeated-measures, zero-migration study using a porcine cadaver tibial implant scanned in 7 positions (21 double examinations). Migration was reported as maximum total point motion (MTPM) for RSA and asCTRSA. CTMA provided maximum total translation (mTT) as an estimate of MTPM. Mean rigid-body fitting error (ME) was calculated for RSA and asCTRSA (acceptability threshold 0.35 mm). The minimal important difference for MTPM/mTT was 0.1 mm. asCTRSA was applied to 1 clinical case with known migration 3 and 8 months postoperatively (NCT04017533).

**Results:**

In the porcine study, precision was 0.09 mm (95% confidence interval [CI] 0.02–0.18) for asCTRSA, 0.08 mm (CI 0.03–0.12) for CTMA, and 0.45 mm (CI 0.20–0.69) for RSA. In the clinical case, asCTRSA detected increasing migration (MTPM 2.5–3.6 mm) and exceeded CTMA’s mTT by 0.4 mm at 8 months. Mean rigid-body fitting error remained below 0.35 mm.

**Conclusion:**

We showed that asCTRSA enables automated, surface-based CT-RSA and demonstrated higher precision than RSA in this zero-migration cadaver setting.

Understanding and quantifying implant migration can serve as an indicator of later revision due to loosening in arthroplasties [1]. Marker- and model-based RSA are commonly used research methods for precise measurement of implant migration in different joints [2-4]. RSA calculates rigid-body motion of implants between a baseline and follow-up image by calculating translations and rotations relative to the insertion bone [5]. Despite its precision, RSA’s clinical use is limited by the need for invasive tantalum beads insertion, specialized and high-cost equipment, and trained personnel [6].

CT-based RSA (CT-RSA) is a general term to describe a non-invasive alternative that uses broadly accessible CT infrastructure to calculate implant migration by comparing 2 sequential CT scans [2]. Recent studies demonstrate that CT-RSA provides comparable precision to traditional RSA methods, particularly when modern CT protocols with metal artifact reduction and sub-millimeter resolution are employed [7-9]. Typically, CT-RSA methods relied on the manual placement of virtual anatomical landmarks (CT-based Micromotion Analysis, CTMA v25.1, 2023, Lidköping, Sweden) to estimate implant migration [10]. More recently, an updated CTMA implementation beyond the version used in this study, and V3MA [11], now perform a surface-based or volume-based analysis. However, these approaches require minor manual steps that may introduce user dependence and lack quality measures for result reliability. An AI-based CT-RSA has been proposed as a proven user-independent approach, but it remains landmark-based and also does not report quality measures [12].

In our study, we present a novel, automated, surface-based CT-RSA method (asCTRSA) that incorporates an objective quality measure to quantify the reliability of the migration analysis. The aim was to evaluate the precision of migration measurements derived from asCTRSA by comparing it with marker-based RSA (RSA) and CTMA in both a porcine cadaver model with zero migration and a patient case with known migration.

## Methods

### Study design

The performance of asCTRSA was evaluated in 2 settings: a repeated-measures study on a porcine cadaver with a tibial implant under zero-migration conditions, and a patient case with confirmed implant migration assessed at 3 and 8 months postoperatively (ClinicalTrials.gov NCT04017533). In both settings, asCTRSA was compared against 2 reference methods: marker-based RSA and CTMA (v25.1). Migration was quantified as maximum total point motion (MTPM) for asCTRSA and RSA, and as maximum total translation (mTT) for CTMA, an estimate of MTPM. Differences between methods were evaluated against a minimal important difference (MID) of 0.1 mm, consistent with previous CT-based precision studies [13,14]. The study is reported according to STARD guidelines [15].

### Pipeline for implant migration analysis

**Figure 1:**
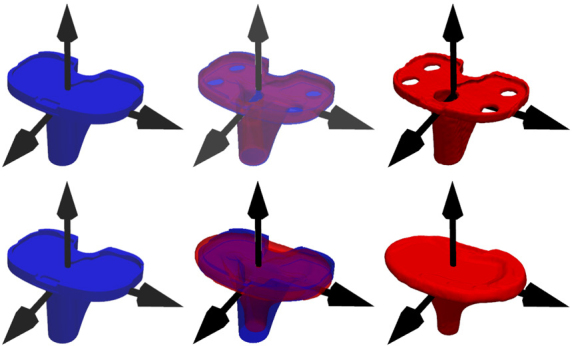
Principal component analysis (PCA) applied to a CAD model (blue) to determine its anatomically meaningful principal axes (black). Subsequently, an implant from a new patient (red) can be aligned to this CAD model using iterative closest point (ICP) registration. This alignment enables automatic extraction of a coordinate system for implants with sufficiently distinctive geometry. The x-, y-, and z-axes correspond to the medial, anterior, and proximal directions, respectively.

Our proposed asCTRSA method quantifies implant migration between baseline and follow-up CT scans with the following automated steps:

1. Segmentation of the tibial bone and the tibial implant in baseline and follow-up images using Otsu thresholding [16] and morphological operations, followed by automatic surface mesh generation.
2. Definition of an anatomically meaningful coordinate system for implant surface mesh on baseline image, with positive translations in a right knee as medial (x-axis), anterior (y-axis), and proximal (z-axis), and corresponding positive rotations as transverse (x), varus (y), and internal (z). Principal axes were computed via principal component analysis (PCA) on the computer aided design (CAD) model surface mesh (Figure 1). The surface vertices of the CAD model were then rigidly aligned using iterative closest point (ICP) [17] to the surface vertices of the implant mesh derived from the CT image (step 1). This allows the automatic registration of anatomically meaningful axes for other implant shapes with sufficiently distinctive geometry.
3. Rigid pair-wise image registration to obtain the optimal transformations (T_tibia_ and T_implant_) to align the tibia and implant segmentations of baseline and follow-up images using intensity-based similarity metrics. The registration method is based on a previously published pipeline [18].
4. Determination of relative motion between the separate alignments can detect implant migration.
$$T_{\text{relative}}=T_{\text{implant}}\times T_{\text{tibia}}^{-1}$$
5. Total translation is defined as the Euclidean distance between corresponding surface vertices a_n_ on the baseline image and their transformed positions ${\text{a}}_{\text{n}}^{\text{t}}$ after applying the relative motion T_relative_.
$$TT_{\text{n}}=\left|a_{\text{n}}-a_{\text{n}}^{\text{t}}\right|,\;\text{n}=1,\ldots ,N$$
  where n is a vertex index on the implant surface and TT is calculated for the position of this vertex a_n_ and N is the amount of surface vertices.
6. Calculation of maximum total point motion (MTPM) as the maximum total translation (TT) over the n surface vertices.
$$\text{MTPM}=\underset{\text{n}=1,\ldots ,\text{N}}{\max}TT_{\text{n}}$$
7. Visualization of implant migration using 1) a heatmap showing the range of total TT over the implant, 2) the location of the point with maximum motion, and 3) the direction in which that point migrated.

Our novel Python-based migration analysis method (asCTRSA) is fully automated and requires only 2 CT volumes as input, with no additional user interaction. Each processing step (1, 2, 3, and 7) produces outputs that can be visually inspected to identify potential errors. asCTRSA was compared with 2 reference methods: CT-based micromotion analysis (CTMA) and marker-based RSA (RSA), both previously described by Engseth et al. [19].

**Figure 2:**
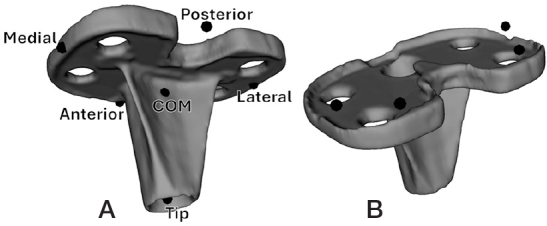
(A) In the CTMA method, total translation (TT) is calculated for 6 manually placed virtual landmarks: anterior, posterior, medial, lateral, center of mass (COM), and tip. (B) For marker-based RSA (RSA), 4 tantalum beads are placed in the bearing component secured to the tibial baseplate.

CTMA (version 25.1) was used in its virtual landmark-based workflow. In brief, CTMA calculated migration for 6 manually placed virtual implant landmarks (anterior, posterior, medial, lateral, center of mass, tip; Figure 2). The highest TT among these is an estimate of the MTPM derived from the full implant set and is referred to as maximum total translation (mTT) [13]. This method includes several manual steps, including semi-automatic image registration and landmark placement for both anatomical axes (step 2) and total translation calculation (step 5). In contrast, asCTRSA automates the process and evaluates migration for all surface vertices.

RSA was performed using the RSAcore 4.2 (LUMC, Leiden, the Netherlands) and served as gold standard for implant migration analysis.

### Preclinical animal model and patient study

The zero-motion porcine cadaver dataset was originally acquired by Engseth et al. [19]. The dataset comprised 7 biplanar radiographs and 7 CT images of a single porcine specimen implanted with a NexGen CR total knee prosthesis, including a tibia C, 10-mm insert, and femur size 4 component (Zimmer Biomet, Warsaw, IN, USA). 10 tantalum markers (Ø 1 mm; RSA Biomedical, Umeå, Sweden) were inserted into the tibia, and 4 were placed in the polyethylene component of the implant in line with marker-based RSA guidelines [2]. The polyethylene insert was frozen when we performed the images. We therefore consider the insert–implant structure as a fixed compound without movements in between.

Biplanar radiography and CT scans were acquired following each of 7 distinct positions of the cadaver (P1 through P7), resulting in 21 double examinations (P1 vs P2, P1 vs P3, …, P5 vs P7, P6 vs P7), where the first position in each pair served as the baseline image and the second as the follow-up. Biplanar radiographs (133 kV and 6.3 mAs) were acquired with the Proteus XR/A (GE Healthcare, Chicago, IL, USA) and Canon Triathlon T3 (Canon, Tokyo, Japan). CT scans were acquired with a revolution system (GE Healthcare, USA) using the following acquisition parameters: 120 kV, 100 mAs, 0.625 mm slice thickness, pitch 1.0, and 200×200 mm field-of-view. The mean effective radiation doses were 0.005 mSv for the RSA and 0.08 mSv for CT. The acquired imaging series was reconstructed using ASiRV50 and MAR. These scans were analyzed using asCTRSA and the reference CTMA method. Migration was quantified using standardized RSA parameters: TT and MTPM or mTT [10]. A group-average rise of MTPM over 0.2 mm from year 1 to 2 after surgery may signal early loosening [20], underscoring the need for high precision measurements.

A first patient case was analyzed based on known migration. The patient (54F) underwent total knee arthroplasty with a Medacta GMK Sphere implant (Medacta International, Castel San Pietro, Switzerland). She experienced early postoperative loosening without an identifiable underlying cause. Migration was assessed between the baseline scan and follow-up scans at 3 and 8 months postoperatively for both biplanar radiographs and CT imaging. Mean effective radiation doses were 0.02 mSv for RSA and 0.07 mSv for CT. No clinical events or interventions that could affect implant stability occurred between imaging timepoints. All migration analyses were performed independently and without knowledge of migration results of the other methods.

### Quality parameters

Similar to RSA, asCTRSA outputs a quality measure; mean error of rigid-body fitting (ME).

The error and its threshold are calculated according to the standard ISO 16087:2013(E) adjusted as explained below [21]. For RSA, mean rigid-body fitting error is defined as the mean Euclidean distance between the beads in the baseline and follow-up scans after registration, with values below 0.35 mm considered acceptable. For asCTRSA, mean rigid-body fitting error was calculated point by point for the implant mesh as the mean Euclidean distance between implant vertices of the baseline and follow-up segmentations after intensity-based registration. Point-by-point correspondence was established using iterative closest point (ICP) [17] to rigidly align the baseline and follow-up segmentation.

### Statistics

**Figure 3:**
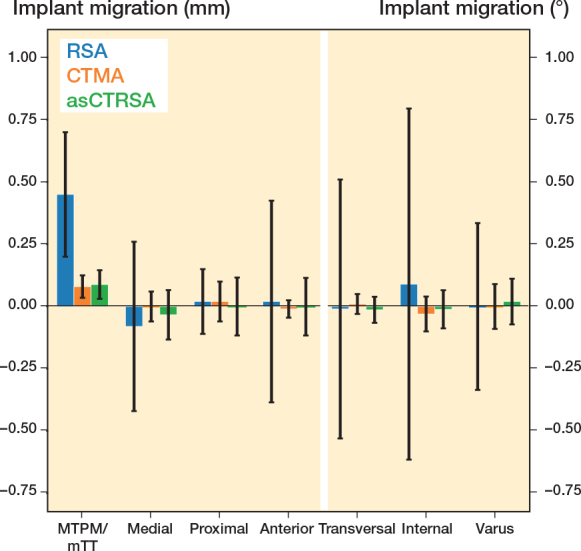
The precision of asCTRSA (green) compared with the 2 reference methods marker-based RSA (RSA, blue) and CTMA (yellow) and visualized using mean and 95% confidence interval. The mean migration over all implant surface points is defined in 6 DOF: 3 translations (medial, proximal, anterior) and 3 rotations (transversal, internal, varus). For RSA and asCTRSA, MTPM refers to the point with maximum motion derived from the full implant surface. CTMA calculates migration for a set of 6 landmarks and can only estimate MTPM using maximum total translation (mTT).

This exploratory study evaluates the precision of asCTRSA against 2 reference methods, CTMA and RSA, using linear mixed-effect modelling. The outcome variable was MTPM (for asCTRSA and RSA) or mTT (for CTMA) for 21 double examinations. This model was established in R (version 4.5.1; R Core Team, 2025; R Foundation for Statistical Computing, Vienna, Austria) using the nlme package. The type of method (asCTRSA, CTMA, RSA) was included as a fixed effect, as between-method differences are the primary effect of interest. Double examination was modelled as a random intercept, to separate the effect of correlation among measurements within the same double examination from the fixed effect of method. This implies compound symmetry, meaning equal covariance between methods within the same double examination. The unequal variance across methods was evident (see Figure 3) and modelled with a heteroscedastic model by allowing each method its own residual variance (varIdent weighting structure). Model parameters were estimated by restricted maximum likelihood (REML). Degrees of freedom (df) were estimated using the containment method, yielding df = 40. Estimated marginal means and 3 pairwise contrasts (RSA vs asCTRSA, RSA vs CTMA, and asCTRSA vs CTMA) were obtained using the emmeans package.

Results are given as least-squares means and between-method differences, each with 95% confidence intervals (CI: ± 1.96*SD).

### Ethics, registration, data sharing plan, funding, and disclosures

Both the porcine cadaver and the single patient had existing ethical approvals. For the porcine study, the Regional Committees for Medical and Health Research Ethics (REK, Norway) approved the study on December 13, 2021. For the patient, the same committee approved the study (reference 2014/1075/REK Vest) from which 1 convenience case was included in the present analysis. The protocol is available at ClinicalTrials.gov (NCT04017533). Written consent from the patient was obtained preoperatively. This study was supported by an IRP project funded by Vrije Universiteit Brussel (VUB, Brussels, Belgium). No conflicts of interest are reported and data can be obtained upon reasonable request. Complete disclosure of interest forms according to ICMJE are available on the article page, doi: 10.2340/17453674.2026.46156

## Results

### Porcine cadaver with no implant migration

In this dataset, implant migration is known to be zero. Thus, all migration measurements reflect measurement error under zero-motion conditions. The MTPM was 0.45 mm (CI 0.20–0.69) for the conventional RSA method. mTT by CTMA was 0.08 mm (CI 0.03–0.12) and MTPM by asCTRSA was 0.09 mm (CI 0.02–0.18) (Table 1, Figure 3).

**Table 1:** Translation and rotation around the anatomical axes using asCTRSA compared with marker-based RSA (RSA) and CTMA, reported as mean and 95% confidence interval

Item	RSA	CTMA	asCTRSA
MTPM/mTT (mm)	0.45 (0.20 to 0.69)	0.08 (0.03 to 0.12)	0.09 (0.02 to 0.18)
Medial (mm)	–0.08 (–0.42 to 0.27)	0.00 (–0.06 to 0.06)	–0.03 (–0.13 to 0.07)
Proximal (mm)	0.02 (–0.11 to 0.15)	0.02 (–0.06 to 0.10)	–0.00 (–0.12 to 0.11)
Anterior (mm)	0.01 (–0.40 to 0.41)	0.01 (–0.03 to 0.04)	–0.00 (–0.12 to 0.12)
Transversal (°)	–0.01 (–0.51 to 0.53)	0.01 (–0.03 to 0.05)	–0.01 (–0.06 to 0.04)
Internal (°)	0.09 (–0.62 to 0.80)	–0.03 (–0.10 to 0.04)	-0.01 (–0.09 to 0.07)
Varus (°)	0.00 (–0.34 to 0.34)	0.00 (–0.09 to 0.09)	0.02 (–0.07 to 0.11)

Difference in mTT computed by CTMA and MTPM computed by RSA was 0.36 mm (CI 0.13–0.61) (Table 2). Similarly, the difference in MTPM between asCTRSA and RSA was 0.36 mm (CI 0.12–0.60). No statistically significant difference was observed between the CT-RSA methods, with asCTRSA showing higher values (0.01 mm, CI –0.01 to 0.03).

**Table 2:** Estimates from the linear mixed model with the RSA method as the reference method, compared with asCTRSA and CTMA

Difference	Estimate (mm)	SE (mm)	df
RSA–asCTRSA	0.36	0.03	40
RSA–CTMA	0.37	0.03	40
asCTRS–CTMA	0.01	0.01	40

df = degrees of freedom

### Quality metrics

**Figure 4:**
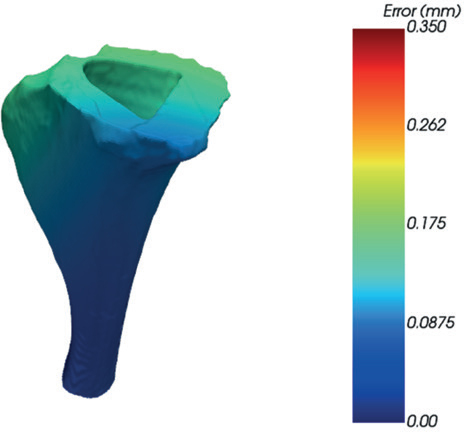
Mean error of rigid-body fitting (ME) visualized over the entire bone shape. Following the standard ISO 16087:2013(E) for RSA migration analysis, any value smaller than 0.35 mm is deemed acceptable.

Quality metric mean rigid-body fitting error was calculated for the porcine data (Table 3). For the tibia, asCTRSA produced slightly smaller values than RSA. A similar pattern was observed for implant registration, with both methods yielding slightly lower values than for the tibia. All errors calculated were below the limits set for RSA, i.e., 0.35 mm. As mean rigid-body fitting error is calculated point by point for all surface vertices of the bone and implant mesh, the error can be visualized using a heatmap (Figure 4).

**Table 3:** Quality measure mean error of rigid-body fitting (ME) with 95% confidence interval (CI) for asCTRSA and RSA

Location	RSA, ME (CI), mm	asCTRSA, ME (CI), mm
Tibia	0.12 (0.01–0.23)	0.07 (0.04–0.10)
Implant	0.11 (0.02–0.20)	0.03 (0.02–0.04)

### Patient case

**Figure 5:**
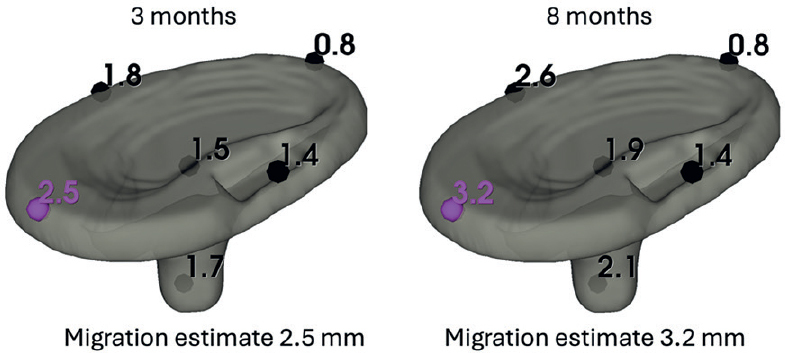
Total translation (TT) for 6 manually placed landmarks using CTMA. MTPM is then calculated as the maximum TT of this set. Migration was analyzed for both 3 months and 8 months post-surgery.

**Figure 6:**
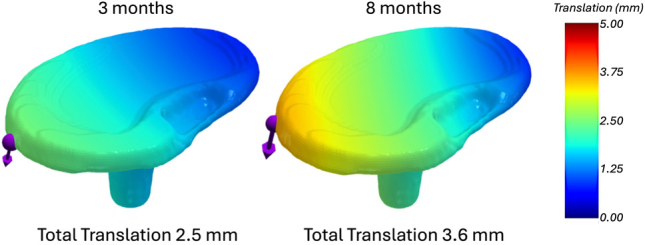
Total translation using asCTRSA, visualized using a heatmap for a first patient case. Migration was analyzed both 3 months and 8 months post-surgery.

MTPM of asCTRSA increased from 2.5 mm at 3 months to 3.6 mm at 8 months post-operation (Table 4). MTPM by RSA increased from 2.3 mm to 3.5 mm. With CTMA, the medial landmark shows the highest migration (mTT) for both follow-up images (Figure 5), increasing from 2.5 mm to 3.2 mm. In contrast, asCTRSA identifies MTPM at a medial location after 3 months, which shifts more anteriorly after 8 months (Figure 6).

**Table 4:** Migration in a patient with known migration: the asCTRSA method compared with RSA and CTMA

Follow-up	asCTRSA (MTPM), mm	RSA (MTPM), mm	CTMA (mTT), mm
3 months	2.5	2.3	2.5
8 months	3.6	3.5	3.2

Quality parameter mean rigid-body fitting error was calculated for both tibial bone and tibial implant (Table 5). Values remained below the RSA-defined threshold of 0.35 mm. The error is higher after 8 months than after 3 months for both bone and implant.

**Table 5:** Quality measure mean error of rigid-body fitting (ME) in a patient with known implant migration using asCTRSA

Location	Follow-up, months	ME, mm
Tibia	3	0.05
	8	0.08
Implant	3	0.12
	8	0.27

## Discussion

This is the first report of fully automated surface-based software with quality measure that does not require user input or manually placed landmarks.

In this study, we compared the precision of an automated surface-based CT-RSA method (asCTRSA) against marker-based RSA and landmark-based CTMA in both a porcine cadaver model under zero-migration conditions and a patient case with known implant migration.

In the porcine cadaver, any measured MTPM reflects method error rather than true migration. We showed that the RSA measurement error exceeded the MID of 0.1 mm, indicating clinically relevant bias, while both CT-RSA methods remained below this threshold. The difference between the 2 CT-RSA methods is not statistically significant (0.01 mm, CI 0.00–0.01) and not clinically relevant, as it remains below the MID of 0.1 mm.

Previous studies found the precision of CT-based methods comparable or superior to RSA [9,12,19]. Hext et al. [8] reported higher precision for CT-RSA over model-based RSA in total knee arthroplasty. While RSA remains the reference standard, differences in reported migration raise questions about which method best reflects actual implant motion. Engseth et al. [7] observed lower migration values with CTMA compared with RSA. However, Angelomenos et al. [22] suggested that CT-RSA and RSA are clinically equivalent. If CT-based methods prove more precise and accurate, existing clinical thresholds for unstable migration may need reconsideration.

Mean rigid-body fitting error remained within accepted RSA thresholds for both tibia and implant (< 0.35 mm). As asCTRSA evaluates all surface vertices rather than a limited set of beads, CT-specific thresholds for mean rigid-body fitting error (ME) may differ from those defined for RSA and warrant future investigation.

Conventional RSA also reports the condition number (CN) to quantify collinearity of the tantalum bead distribution (threshold: 120 m^–1^). High values may occur when a limited bead configuration poorly represents overall implant motion. However, for CT-based migration analysis this metric is not relevant because the full implant surface is used, making collinearity unlikely and values typically far below the RSA threshold. In the future, a CT-specific shape metric can be introduced that evaluates geometric symmetry of the bone or implant shape. This can for example be based on (i) the minimum self-distance under small rigid perturbations [23], or (ii) an ISS-style criterion of the local scatter matrix [24].

In the patient case, RSA, CTMA, and asCTRSA detected increasing migration from 3 to 8 months (see Table 4). At 8 months’ follow-up, CTMA yields a lower mTT than the full-surface MTPM calculated by asCTRSA (3.2 mm instead of 3.6 mm). asCTRSA detected a positional shift of MTPM that was not captured for mTT by CTMA, likely because the full-surface approach of asCTRSA is inherently less constrained than landmark-based methods in detecting such changes, although the true MTPM position is unknown. Previous work suggests that CTMA may report lower migration values than RSA [7]. Our single patient case finds a similar pattern for CTMA, but not for asCTRSA. However, this comparison involves different migration metrics (mTT vs MTPM), which may partly explain the observed differences.

### Limitations

Although a single cadaver enabled controlled comparison between methods, these findings reflect performance under zero-motion conditions and cannot be generalized to the broader patient population. The cadaver setting lacks the variability of clinical conditions such as bone density, metal artifacts, soft tissue interference, patient motion, and different implant shapes. Further research should investigate the method’s robustness and the difference in accuracy between the migration analysis methods using clinical data. Although the automated software supports visual inspection of each processing step and applies predefined thresholds for an objective quality measure, the software currently has limited error handling and might require manual reprocessing.

### Conclusion

Fully automated surface-based asCTRSA showed a precision superior to RSA in a zero-migration cadaver setting and introduces user independence and an objective quality metric into a previously subjective process. Future studies should investigate clinical accuracy and the potential need to redefine migration thresholds for CT-based methods.

## Notes

MA: conceptualization, methodology, software, formal analysis, investigation, visualization, statistical analysis, writing—original draft. BK: conceptualization, methodology, supervision, data interpretation, writing—review and editing. LE: conceptualization, data curation, investigation, patient recruitment, ethics approval, writing—review and editing. FDØ: conceptualization, data curation, resources, investigation, writing—review and editing. GVG: conceptualization, supervision, writing—review and editing, funding acquisition. SMR: conceptualization, data curation, resources, investigation, data interpretation, patient recruitment, ethics approval, writing—review and editing. AS: conceptualization, data interpretation, dose calculations, writing—review and editing. JDM: conceptualization, funding acquisition, project administration. NB: conceptualization, methodology, supervision, writing—review and editing, funding acquisition.

The authors would like to thank Dr Wilfried Cools for his assistance with the statistical analysis and for proofreading the paragraph on the statistical analysis.

Handling co-editors: Bart Pijls and Jonas Ranstam

*Acta* thanks Olof Sandberg and other anonymous reviewers for help with peer review of this manuscript.
